# The Biosynthesis, Structure Diversity and Bioactivity of Sterigmatocystins and Aflatoxins: A Review

**DOI:** 10.3390/jof10060396

**Published:** 2024-05-31

**Authors:** Wenxing Li, Zhaoxia Chen, Xize Li, Xinrui Li, Yang Hui, Wenhao Chen

**Affiliations:** 1 Key Laboratory of Tropical Medicinal Resource Chemistry of Ministry of Education, College of Chemistry and Chemical Engineering, Hainan Normal University, Haikou 571158, China; wenxing2256@163.com (W.L.); 17689849894@163.com (Z.C.); 18343178068@163.com (X.L.); 18735053789@163.com (X.L.); 2 Key Laboratory of Tropical Medicinal Plant Chemistry of Hainan Province, College of Chemistry and Chemical Engineering, Hainan Normal University, Haikou 571158, China

**Keywords:** sterigmatocystins, aflatoxins, structural features, synthesis, biological activity

## Abstract

Sterigmatocystins and aflatoxins are a group of mycotoxins mainly isolated from fungi of the genera *Aspergillus*. Since the discovery of sterigmatocystins in 1954 and aflatoxins in 1961, many scholars have conducted a series of studies on their structural identification, synthesis and biological activities. Studies have shown that sterigmatocystins and aflatoxins have a wide range of biological activities such as antitumour, antibacterial, anti-inflammatory, antiplasmodial, etc. The sterigmatocystins and aflatoxins had been shown to be hepatotoxic and nephrotoxic in animals. This review attempts to give a comprehensive summary of progress on the chemical structural features, synthesis, and bioactivity of sterigmatocystins and aflatoxins reported from 1954 to April 2024. A total of 72 sterigmatocystins and 20 aflatoxins are presented in this review. This paper reviews the chemical diversity and potential activity and toxicity of sterigmatocystins and aflatoxins, enhances the understanding of sterigmatocystins and aflatoxins that adversely affect humans and animals, and provides ideas for their prevention, research and development.

## 1. Introduction

Mycotoxins are a class of small molecular compounds produced by the secondary metabolism of fungi that cause toxic reactions, and there are more than 400 formally defined mycotoxins until 2006 [1]. Mycotoxins are toxic secondary metabolites synthesised by specific fungi belonging to *Aspergillus*, *Alternaria*, *Fusarium* and *Penicillium* species. These mycotoxins have the potential to contaminate produce at various points in the food supply chain [2,3,4]. *Aspergillus* mycotoxins, including sterigmatocystin (STC) and aflatoxin (AF), are the most important mycotoxins [5,6].

Sterigmatocystins (STCs) and Aflatoxins (AFs) are a class of compounds with similar chemical structures and are mycotoxins produced by *Aspergillus* [7], which are widely distributed in nature and are common contaminants in human food as well as animal feed [8,9]. AFs are a specific group of mycotoxins primarily produced by toxigenic *Aspergillus* species, particularly *A. flavus* and *A. parasiticus* [2]. STCs are produced by several fungal species belonging to the genera *Aspergillus*, *Bipolaris*, *Botryotrichum*, *Humicola* and *Penicillium*. The main producers are *Aspergillus* fungi, such as *A. flavus*, *A. parasiticus*, *A. nidulans* and *A. versicolor* [10,11]. These two classes of compounds have characteristic bifuran ring structural fragments, which is the active moiety of these two classes of compounds. These compounds have received extensive attention from researchers due to their abundant sources and diverse biological activities.

This reviews the current research progress in the isolation of STCs and AFs, including the biosynthetic pathway, source of isolation, structure identification process, chemical synthesis, and biological activity. SciFinder was used as the main search engine, and sterigmatocystins and aflatoxins were used as keywords to collect information about the chemical structure, biological activity, and synthesis of these two classes of compounds from 1954 to April 2024. In the study of bioactivity, the main components are antibacterial, anti-inflammatory activities and antitumour mechanisms.

## 2. Biosynthesis, Structural and Biological Activity Studies

### 2.1. Biosynthesis

**Figure 1:**
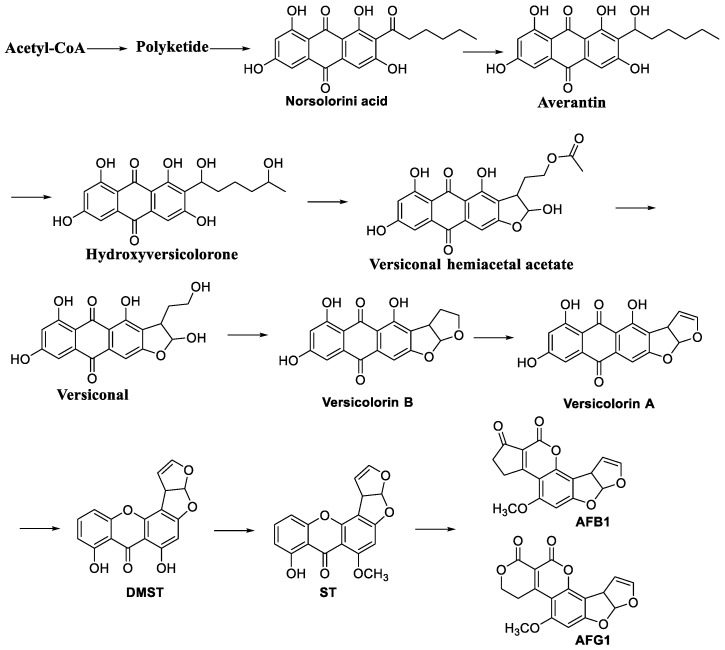
Outline of the biosynthetic pathway of STCs and AFs.

The STCs and AFs are produced from acetyl coenzyme A through a complex branching pathway involving more than 25 enzymatic reactions. The outline of the proposed pathway is shown in Figure 1 [12].

#### 2.1.1. Biosynthesis of Sterigmatocystins

As early as 1968, Holker et al. [13] suggested a possible biological pathway for STC. In 1989–1990, Bhatnagar et al. [14] basically determined the synthesis pathway of STC in the bacterium with the help of radiotracer, gene conversion and metabolism inhibitor experiments. Acetic acid or acetate → desmethylsulphanilic acid → averantin → odoriferous flunaricin → heterochromatic trichothecene hemiacetal acetate → heterochromatic trichothecene A → STC.

**Figure 2:**
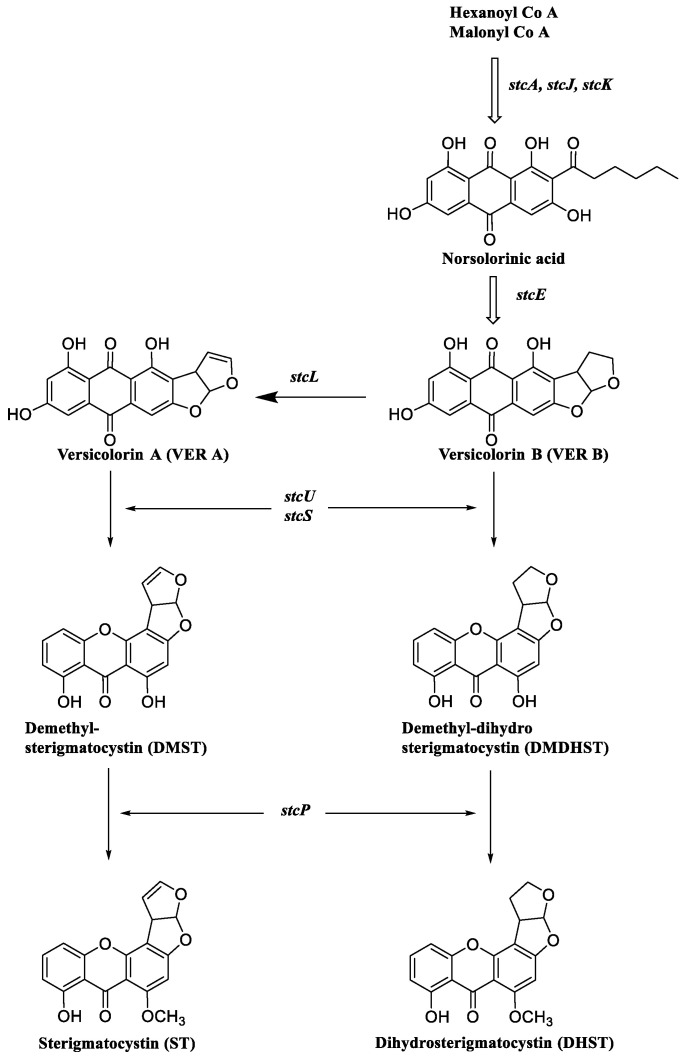
Biosynthesis pathway of STCs [16,17,18].

In 2014, Alkhayyat et al. [15] found that aflR and aflS genes are adjacent in the aflatoxin/sterigmatocystin cluster and are involved in the regulation of aflatoxin/sterigmatocystin gene expression and that mutational inactivation of aflR abolished the expression of any biosynthetic genes in the cluster, and conversely, overexpression of aflR resulted in higher gene expression and AF/STC production (Figure 2).

#### 2.1.2. Biosynthesis of Aflatoxins

**Figure 3:**
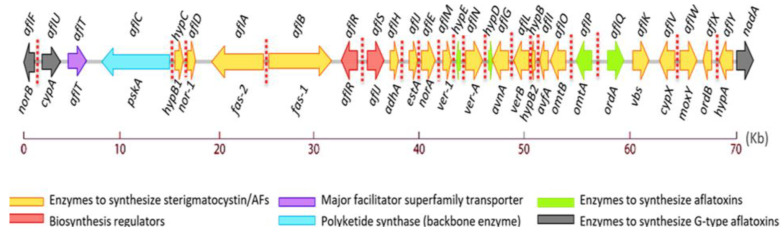
Organisation of gene clusters of AFs biosynthesis pathway [22].

Molecular studies have shown that the synthesis of AFs is regulated by a complex cascade of transcriptional and post-transcriptional processes [19]. The biosynthesis of AFs involves a series of enzymatic and biotransformation reactions, as shown in Figure 3. The pathway is initiated by acetyl-CoA and malonyl-CoA to form hexyl-CoA, which is then further converted to produce norsolorinic acid [2]. The reduction process is facilitated by enzymes such as the gene aflF (norB) and dehydrogenase and NOR reductase encoded by the aflE (norA) gene, respectively. Another important enzyme, HypC, oxidises an intermediate to produce anthraquinone orthoformate (NA). In the third step, the gene aflG (avnA) encodes cytochrome P450 monooxygenase, which catalyses the hydroxylation of polyketide anthraquinone to an intermediate called 5′-oxoanthraquinone (OAVN) [19].

This conversion requires alcohol dehydrogenase encoded by the aflH (adhA) gene. The conversion of versicolorin B and OAVN to AVF (AVF) is attributed to the aflK (vbs) gene, which was initially associated with the conversion of versicolorin B to AVF [2,19,20]. AflV encodes the enzyme responsible for the conversion of AVF to vertical hemiacetal acetate (VHA). Subsequent steps include the conversion of VHA to versiconal (VAL) and versicolorin B (VER B) to versicolorin A (VER A) by specific enzymes. VER A is important because it represents the last metabolic conversion before the major branch, leading to the production of B- or G-type Afs (Figure 3) [2,21].

In the next step, DMST is converted to STC by the action of *O*-methyltransferase B encoded by the aflO (omtB) gene. DHST is converted to dihydromethylsterocystin (DHOMST) and ST to *O*-methylsterocystin (OMST) by the action of *O*-methyltransferase A encoded by the aflP (omtA) gene. OMST and DHDMST are further converted to AFB1/AFG1 and AFB2/AFG2, respectively, by cytochrome P450 monooxygenases encoded by the aflQ (ordA) gene [22,23]. AFB1 is a precursor of OMST, and the hypD gene encodes integral membrane proteins that are essential to produce AF B1 and AF G1 [20].

### 2.2. Sterigmatocystins

**Figure 4:**
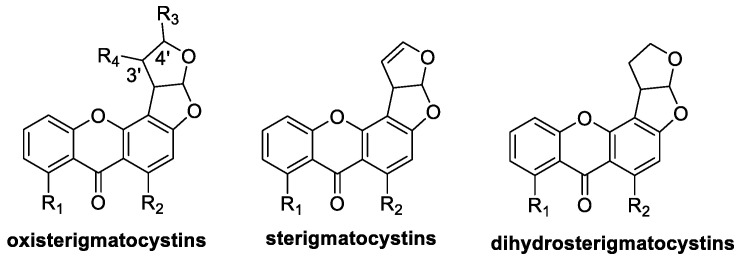
The basic skeleton of Oxisterigmatocystins, Sterigmatocystins, and Dihydrosterigmatocystins.

The STCs are a group of compounds with a bifuranic ring and an anthracenone skeleton, which are widely distributed in nature and are very diverse [24]. STC derivatives are based on STC with one or more substitutions on the bifuranic ring and the aromatic ring, and the main forms of substitution are hydroxylation, alkylation, esterification, halogenation, etc. Depending on the presence or absence of substitutions at their 3′ and 4′ positions and the presence or absence of double bonds, they can be classified into three types, including oxisterigmatocystins, sterigmatocystins, and dihydrosterigmatocystins. The basic skeleton of these three classes of compounds is shown in Figure 4.

#### 2.2.1. Oxisterigmatocystins

**Figure 5:**
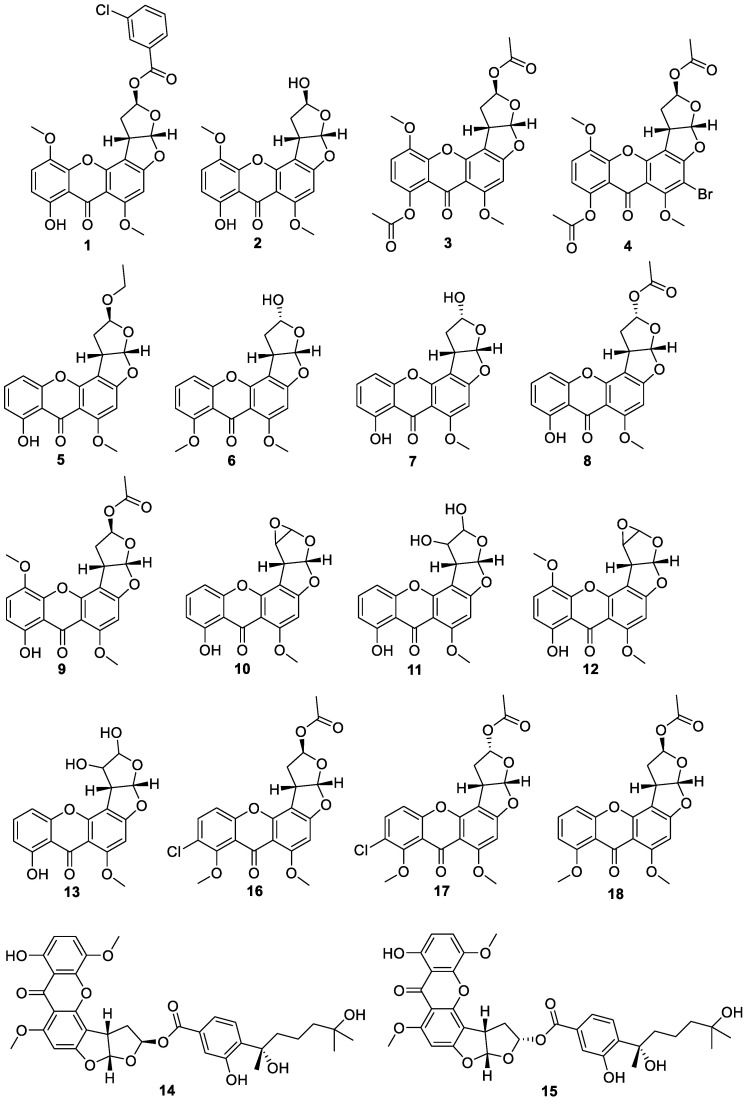

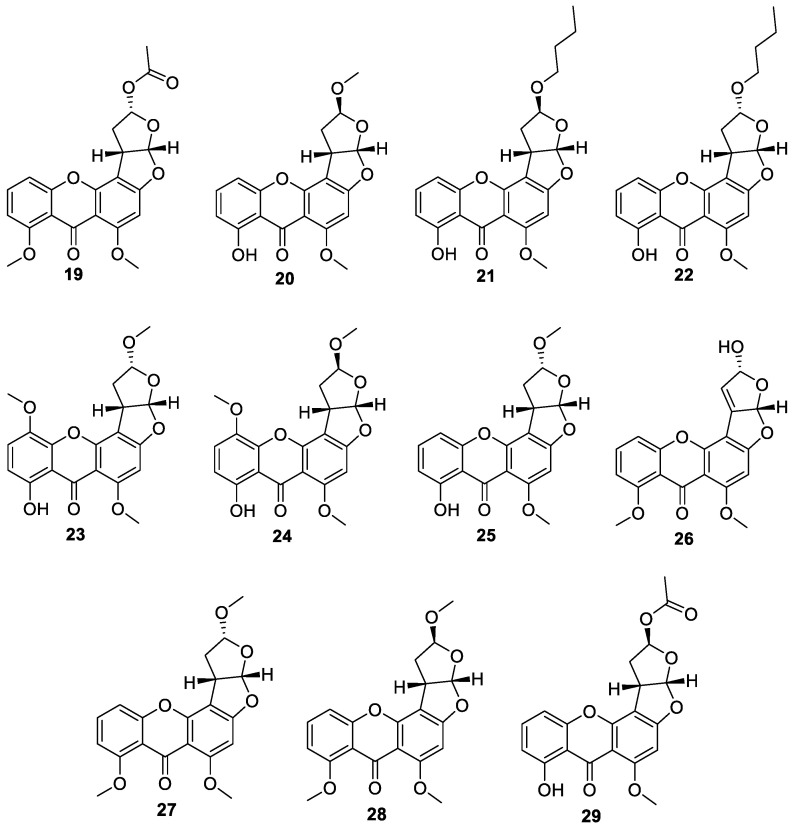
Chemical structures of oxisterigmatocystins **1**–**29**.

Four new oxisterigmatocystins 1,2,3a,12c-tetrabydro-2-(3-chlorobenzoyloxy)-1,8-dihydroxy-6,11-dimethoxy-7*H*-furo[3′,2′:4,5]furo[2,3-c]-xanthen-7-one (**1**), 1,2,3a,12c-tetrabydro-2,8-dihydroxy-6,11-dimethoxy- 7*H*-furo[3′,2′:4,5]furo[2,3-c]-xanthen-7-one (**2**), 1,2,3a,12c-tetrabydro-2,8-diacetoxy-6,11- dimethoxy-7*H*-furo[3′,2′:4,5]furo[2,3-c]-xanthen-7-one (**3**), 1,2,3a,12c-tetrabydro-5-bromo- 2,8-diacetoxy-6,11-dimethoxy-7*H*-furo[3′,2′:4,5]furo[2,3-c]-xanthen-7-one (**4**) (Figure 5) were discovered [25]. Three new compounds, including sterigmatocystin-hemiacetal (**5**), sterigmatocystin-ethoxyacetal (**6**) and *O*-methylsterigmatocystin-hemiacetal (**7**), were isolated from the *Aspergillus* sp. [26]. One new compound, acyl-hemiacetal sterigmatocystin A (**8**), was isolated from *Aspergillus oryzae* M395 of sponge origin [27]. One new compound was isolated from the secondary metabolites of an *Aspergillus* sp. fungus and identified as oxisterigmatocystin D (**9**) [28]. Four new compounds, sterigmatocystin-1,2-oxide (**10**), sterigmatocystin-1,2-dihydrodiol (**11**), methylsterigmatocystin-1,2-oxide (**12**) and methylsterigmatocystin-1,2-dihydrodiol (**13**) in the study of the metabolic pathways of ST and 11-methoxysterigmatocystin [29]. Two new compounds, Aspertenol A (**14**), B (**15**), were isolated from the secondary metabolites of the strain *Aspergillus tennesseensis*. [30]. Four oxisterigmatocystins analogues of oxisterigmatocystins E-H (**16**–**19**) were isolated from secondary metabolites of the fungus *Botryotrichum piluliferum* [31]. A new compound, oxisterigmatocystin I (**20**), was isolated from *Aspergillus versicolor* A-21-2-7 of South China Sea sediments [32]. Five new compounds, sterigmatocystin B (**21**), C (**22**) and oxisterigmatocystin A–C (**23**–**25**), were isolated from *Aspergillus versicolor* 15XS43ZD-1 [33]. Three new compounds, oxisterigmatocystin J–L (**26**–**28**), were isolated from the marine-derived fungus *Aspergillus nomius* NC06 [7,34]. One new compound, acyl-hemiacetal sterigmatocystin B (**29**), was isolated from *Aspergillus nidulans* of Nyctanthes arbor-tristis [35].

#### 2.2.2. Sterigmatocystins

**Figure 6:**
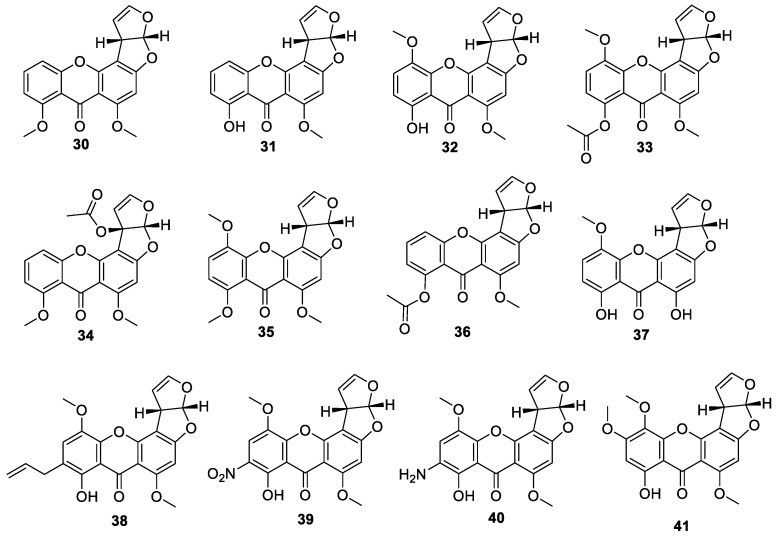

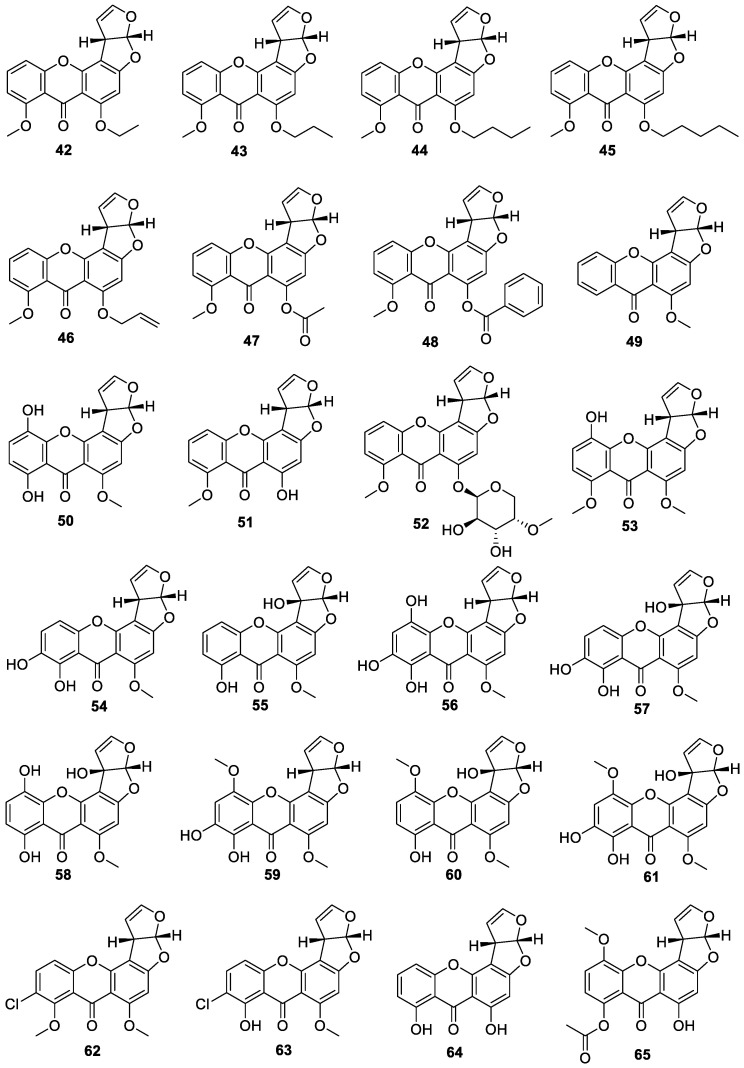
Chemical structures of sterigmatocystins **30**–**65**.

One new compound, *O*-methylsterigmatocystin (**30**), was isolated and identified from the secondary metabolites of the fungus *Aspergillus flavus* [36]. (Figure 6) Three new compounds, including sterigmatocystin (**31**), 5-methoxysterigmatocystin (**32**) and *O*-acetates (**33**), were isolated from a mutant strain of *Aspergillus versicolor* [37]. One new compound, aspertoxin acetate (**34**), was isolated from secondary metabolites of *Aspergillus flavus* [38]. Two new compounds *O*-methyl-5-methoxysterigmatocystin (**35**) and *O*-acetyl-5-methoxysterigmatocystin (**36**), were isolated from *Aspergillus* sp. [39]. Four new compounds, 5-methoxy-*O*-hydroxysterigmatocystin (**37**), 3a,12c-dihydro-9- allyl-8-hydroxy-6,11-dimethoxy-7*H*-furo[3′,2′:4,5]furo[2,3-c]xanthen-7-one (**38**), 3a,12c- dihydro-9-(or10)-nitro-8-hydroxy-6,11-dimethoxy-7*H*-furo[3′,2′:4,5]furo [2,3-c]xanth-en- 7-one (**39**), 3a,12c-dihydro-9-amino-8-hydroxy-6,11-dimethoxy-7*H*-furo [3′,2′:4,5]furo [2,3-c]xanthen-7-one (**40**), were isolated by the activity tracking method to obtain [25]. A new compound, 5,6-dimethoxysterigmatocystin, was isolated and identified from *Aspergillus versicolor* under the guidance of hepatocyte DNA repair assay and *Salmonella* assay (**41**) [40]. Seven new compounds, including *O*-ethylsterigmatocystin (**42**), *O*-propylsterigmatocystin (**43**), *O*-butylsterigmatocystin (**44**), *O*-penlylsterigmatocystin (**45**), *O*-propenylsterigmatocystin (**46**), *O*-acethlsterigmatocystin (**47**), *O*-benzoylsterigmatocystin (**48**), were isolated from *Aspergillus* sp. [41]. One new compound, 8-deoxysterigamoatocystin (**49**), was isolated from the secondary metabolite of the brown-black rotting mould fungus NRRL.22980 [42]. Three new compounds, 5-hydroxysterigmatocystin (**50**), 8-*O*-methoxysterigmatocystin (**51**), demethylsterigmatocystin-1-(4-*O*-methyl)-*β*-L-arabinopyranose (**52**), were isolated from the winged insect endophytic fungus *Aschersonia coffeae* Henn. BCC 28712 [43]. One new compound, 11-hydroxy-*O*-methylsterigmatocystin (**53**), was isolated from *Aspergillus* sp. [44]. Eight new compounds, including 9-hydroxy-sterigmatocystin (**54**), 12c-hydroxy-sterigmatocystin (**55**), 11-hydroxy-sterigmatocystin (**56**), 9,11-dihydroxy-sterigmatocystin (**57**), 9,12c-dihydroxy-sterigmatocystin (**58**), 11,12c-dihydroxy-sterigmatocystin (**59**), 9-hydroxy-methylsterigmatocystin (**60**), 9,12c-hydroxy-methylsterigmatocystin (**61**) have been isolated and identified [29]. Two new halogenated sterigmatocystins, N-0532B (**62**) and N-0532A (**63**), were isolated from the fermentation products. And the fungal strain, *Botryotrichum piluliferum*, was isolated from soil and cultured at 25–28 °C for 9 weeks [31]. A comparative study of the secondary metabolites of the sponge-derived endophytic fungus *Aspergillus carneus* was carried out using the OSMAC method, in which the strains were fermented and cultured in a solid medium with sea-salt rice, solid medium without sea-salt rice and modified Czapek’s medium, and a heterochromatic trichothecene analogue, *O*-demethyld-terigmatocystin (**64**), was isolated from the medium without sea-salt rice [45]. An endophytic fungus, *Aspergillus nidulans*, was isolated from the leaves of the plant (Nyctanthes arbortristis) and its secondary metabolite chemistry was investigated to isolate sterigmatocystin, which was acetylated to give a new heterochromatic trichothecene analogue, *O*-acetyl-sterigmatocystin (**65**) [45].

#### 2.2.3. Dihydrosterigmatocystins

**Figure 7:**
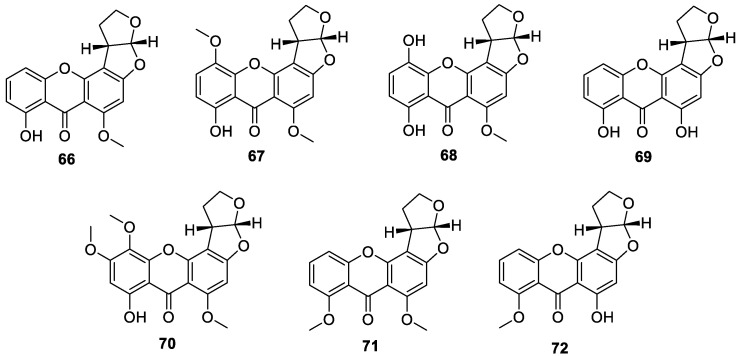
Chemical structures of dihydrosterigmatocystins **66**–**72**.

Isolation of the dihydrosterigmatocystin analogue dihydrosterigmatocystin (**66**) (Figure 7) from a secondary metabolite of *Aspergillus versicolor* and synthesis of the derivatives 5-*O*-methyoxydihydrosterigmatocystin (**67**) and 5-hydroxydihydrosterigmatocystin (**68**) via compound **66** [37]. One new compound, dihydrodemethylsterigmatocystin (**69**), was isolated from *Aspergillus versicolor* [46]. A new compound was isolated and identified as 5,6-dimethoxydihydrosterigmatocystin (**70**) from *Aspergillus versicolor* [40]. One new compound, 11-hydroxy-*O*-methyldihydrosterigmatocystin (**71**), was isolated from *Aspergillus versicolor* [44]. One new compound, 8-*O*-methyldihydrosterigmatocystin (**72**), was isolated from *Aschersonia coffeae* Henn. BCC 28712 [43].

**Figure 8:**
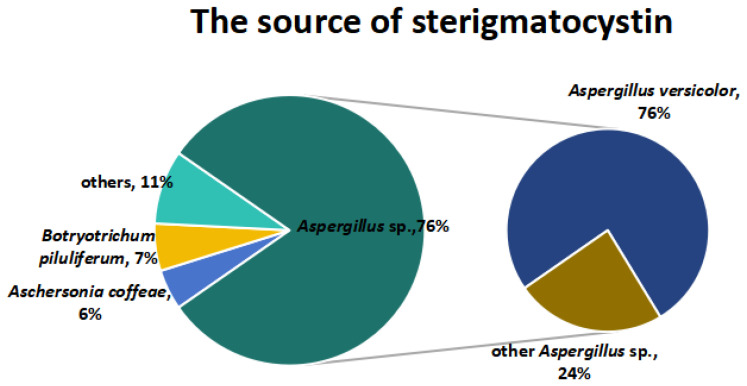
The source of STCs.

The majority of all published STCs isolated and identified in various filamentous fungi were produced by *Aspergillus* sp. (76%), including *Aspergillus versicolor* (76%) and other *Aspergillus* species (24%) (Figure 8).

#### 2.2.4. Activity of Sterigmatocystins

##### Antitumour Activity of Sterigmatocystins

Compound **15** showed better inhibitory activity against K-562 cells [30]. Compounds **16**–**19** showed better inhibitory activity against KB, MCF-7 and NCl-H187 cell lines with IC_50_ values ranging from 0.38–78.6 µM [31]. Compounds **31** and **32** have broad-spectrum antitumour activity. Compounds **31** and **32** showed significant inhibitory effects on transplanted mouse leukaemia P-388 and L-1210 cells, which is the first report of antitumour activity of this class of compounds [39]. In addition, compound **31** exhibited significant cytotoxicity against human solid tumour cell lines (A549, SK-OV-3, SK-MEL-2, XF-498 and HCT-15) with IC_50_ values ranging from 0.41–4.61 µg/mL [47]. Meanwhile, compound **31** showed strong cytotoxicity against L5178Y, BT-549, HepG2, HGC-27, UMUC-3, MCF-7, Hela, HT-29, HCT-116 and other cell lines, with IC_50_ values ranging from 0.3–58 µM [45,48,49,50,51]. There are many studies on the antitumour activity of compound **31**, and the mechanism of action of compound **31** mainly focuses on the induction of the G2 phase block in GES-1 cells. Studies have shown that the activation of the ATM/Chk2 signalling pathway by **31** may be one of the molecular mechanisms by which **31** induces G2 phase block in GES-1 cells and that compound **31** is capable of activating the JNK, ERK, PI3K pathways, etc. [48,51,52,53,54]. Compound **36** showed significant inhibitory effects on transplanted mouse leukaemia P-388 and L-1210 cells [39]. Compounds **50**–**52** showed good cytotoxic activity against NCl-187 cells with IC_50_ values of 1.17, 12.93, and 2.86 µg/mL, respectively. [43]. Compound **64** exhibited some antibacterial activity against *Fusarium oxysporum* [55]. Compound **66** showed strong antimicrobial activity against *Bacillus cereus* with a MIC value of 38.3 µM and weak inhibitory activity against *Fusarium oxysporum* [55,56].

##### Antimicrobial Activity of Sterigmatocystins

Compound **20** showed moderate bacteriostatic activity against *Staphylococcus aureus* ATCC25923 and *Vibrio parahaemolyticus* ATCC17802 [57]. The results of antimicrobial activity studies showed that compounds **31** and **32** showed some antimicrobial activity against *Staphylococcus aureus*, *Bacillus subtilis*, *Pseudomonas aeruginosa*, *Escherichia coli* and *Candida albicans*, with MIC values ranging from 3.06–12.5 µg/mL [27,58]. In addition, **32** has good bacteriostatic activity against *Streptococcus pyogenes*, *Branchiostoma circumcisionale*, and *Micrococcus garciniae,* all with MIC value of 5 µg/mL [59].

##### Anti-Inflammatory Activity of Sterigmatocystins

Compounds **31** and **32** had a significant effect on NO production in their anti-inflammatory activity and a slight inhibitory effect on THF-α and IL-6 expression. Both compounds inhibited NO production by down-regulating the expression of inducible nitric oxide synthase (iNOS) and also reduced the nuclear translocation of NF-κB. These experimental results suggested that these two compounds are promising candidates for the treatment of neuroinflammatory diseases [60].

##### Antiplasmodial Activity of Sterigmatocystins

In the antiplasmodial activity screen against *Plasmodium falciparum*, compounds **16**–**19** were found to possess good antimalarial activity with IC_50_ values of 7.9–23.9 µM [31]. Compound **52** showed good antiplasmodial activity with an IC_50_ value of 4.39 µg/mL [43].

### 2.3. Aflatoxins

AFs are a class of fungal secondary metabolites with similar structural and physicochemical properties and are the stable class of mycotoxins with physicochemical properties found in nature [61]. It is a class of compounds containing a difuran ring and coumarin (oxonaphthoquinone) in their basic structure. It produces blue-violet or yellow-green fluorescence under the wavelength of 365 nm ultraviolet light. There are about 20 AF derivatives, which are named B1, B2, G1, G2, M1, M2, P1, GM and toxinol based on fluorescent colours, RF values and different structures [62]. The four main types of AFs (AFB1, AFB2, AFG1 and AFG2) are the best-known and best-studied of the 20 different types and metabolites identified so far [61].

The discovery of AF came about as a result of the first occurrence of “Turkey X Disease” (the cause of which was unknown at the time, hence the name) in 1960 in a suburb of London, England [63]. In 1961, it was found that peanut cake contaminated with *Aspergillus flavus* could induce liver cancer in rats, and in 1962, AF was identified and proved to be a strong carcinogen. AF, with a double bond at the end of the difuran ring in its structure, is prone to form epoxidation metabolites that enhance its toxicity, carcinogenicity, and mutagenicity [64].

**Figure 9:**
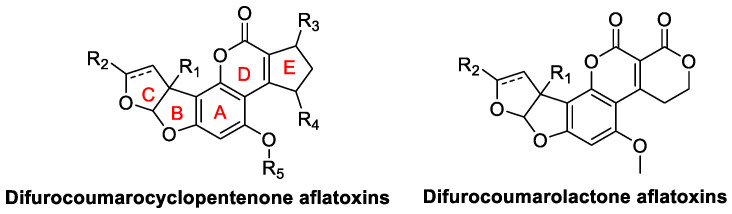
The basic skeleton of Difurocoumarocyclopentenone aflatoxins, Difurocoumarolactone aflatoxins.

Depending on whether the E-ring in the AF structure is a five-membered or six-membered ring, AFs can be classified into two groups: Difurocoumarocyclopentenone aflatoxins, Difurocoumarolactone aflatoxins. The basic skeleton of these two classes of compounds is shown in Figure 9.

#### 2.3.1. Difurocoumarocyclopentenone Aflatoxins

**Figure 10:**
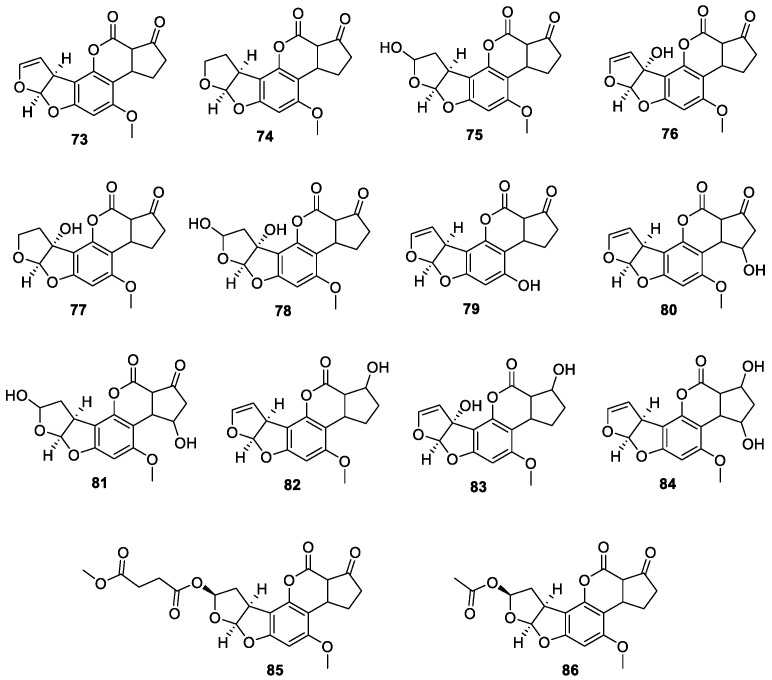
Chemical structures of difurocoumarocyclopentenone aflatoxins **73**–**86**.

There are 14 difurocoumarocyclopentenone aflatoxins in total, namely AF B1 (**73**), AFB2 (**74**), AFB2_a_ (**75**), AFM1 (**76**), AFM2 (**77**), AFM2_a_ (**78**), AFP1 (**79**), AFQ1 (**80**), AFQ2_a_ (**81**), aflatoxicol M1 (**82**), aflatoxicol B1 (**83**), aflatoxicol H1 (**84**), AFB2_b_ (**85**), 8-acetyloxyaflatoxin B1 (**86**) (Figure 10).

In 1964, the structure of AFB1 (**73**) was elucidated [65]. In 1967, the absolute configuration of AFB1 (**73**) and B2 (**74**) was determined by an X-ray single crystal diffraction test [66]. AFB2_a_ (**75**) was discovered while testing the ability of various microbes to detoxify AFB1 [67]. AFM1 (**76**), isolated from AFB1 metabolites hydroxylated by the hepatic microsomal mixed-function oxidase system (MFO), mainly cytochromes, in the liver of mammals [68]. AFM2 (**77**) was isolated from *A. parasiticus* [69]. AFM2_a_ (**78**) was the hydration of the terminal furan ring of AFM1 in dilute acid to yield a hemiketal derivative [70]. AFP1 (**79**) was a demethylated metabolite of aflatoxin B1 by liver microsomal oxidase–catalysed *O*-demethylase [71]. AFQ1 (**80**) was a hydroxylated metabolite of aflatoxin B1 by microsomal enzymes in the liver of higher vertebrates and poultry [72]. AFQ2_a_ (**81**), acid hydration of AFQ1 (**80**) [73]. Aflatoxicol M1 (**82**) was a reduced metabolite of AFB1, AFR0, or AFM1 catalysed by soluble NADPH-dependent reductases in the liver [68]. Aflatoxicol B1 (**83**) a metabolite of AFB1 (**73**) formed by a reversible reduction of the pentanone group in humans, animals and numerous bacteria and moulds [74]. Aflatoxicol H1 (**84**) was a reduced metabolite of AFB1 and AFQ1 (**80**) catalysed by soluble NADPH-dependent reductases in the liver [75]. Two difurocoumarocyclopentenone aflatoxins, AFB2_b_ (**85**) and 8-acetyloxyaflatoxin B1 (**86**), were isolated from the marine-derived fungus *Aspergillus flavus* 092008 [76].

#### 2.3.2. Difurocoumarolactone Aflatoxins

**Figure 11:**
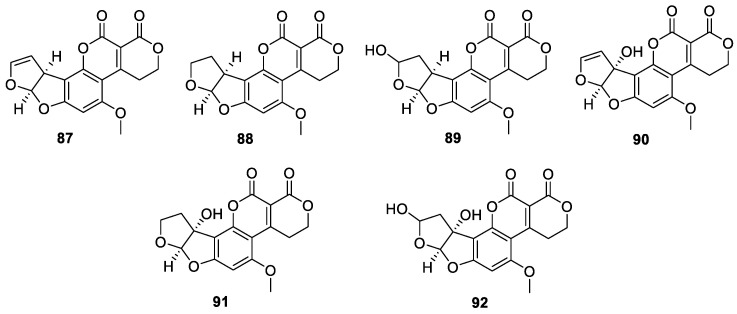
Chemical structures of difurocoumarolactone aflatoxins **87**–**92**.

Difurocoumarolactone aflatoxins have a total of seven compounds, namely AFG1 (**87**), AFG2 (**88**), AFG2_a_ (**89**), AFGM1 (**90**), AFGM2 (**91**), AFGM2_a_ (**92**) (Figure 11).

Two difurocoumarolactone aflatoxins, including AFG1 (**87**) and AFG2 (**88**), were isolated from *A. flavus* [77]. AFG2_a_ (**89**) was a hydroxylated metabolite of AFG1 obtained by catalytic addition of water to the double bond of the terminal furan under acidic conditions in the liver, the stomach or soil [78]. AFGM1 (**90**) was a hydroxylated metabolite of AFG1 by MFO in the liver of mammals [79]. AFGM2 (**91**) was a hydroxylated derivative of AFG2 by MFO in the liver of mammals produced in vitro by *A. parasiticus* from dihydro-*O*-methylsterigmatocystin (DHOMST) [80]. AFGM2_a_ (**92**) was hydration of the terminal furan ring of AFM1 in dilute acid to yield a hemiketal in vitro in liver homogenates [80].

#### 2.3.3. Activity of Aflatoxins

AFB1 (**73**), B2 (**74**) and G1 (**87**) modulate cytokine secretion and cell surface marker expression in J774A.1 mouse macrophages [81]. AFB1 (**73**) has better activity against A549 cells [82]. Liver microsomal metabolites of AFB1 (**73**) induce mutations in DNA repair-deficient bacteria [83]. Compound **73** showed good activity against Hep G2 cells with an IC_50_ value of 16.9 µM. When the cell cycle was arrested at the G0/G1 phase by **73**, the experimental results from the flow cytometry assay demonstrated that the rate of cell apoptosis and mitochondrial membrane potential was also additively increased in a dose-dependent manner. Thus, the integrity of mitochondria (MMP and membrane potential) which was the central component of cell apoptosis, is disrupted by AFB1 and sterigmatocystin in an additive manner. With the immunocytochemistry analysis showing increased expression of apoptosis-related proteins of Bax, Caspase-3 and p53 and decreased expression of Bcl-2 protein, the additive nature of the co-proapoptotic activity of AFB1 was revealed [84,85]. Relaxant effects of AFs: B1, B2, G1 and G2 and their major metabolites (M1, M2, P1, Q1 and G2_a_) on carbachol-preconstricted guinea-pig trachea [86]. AFB2_b_ (**85**) exhibited moderate antimicrobial activity against *Escherichia coli*, *Bacillus subtilis* and *Enterobacter aerogenes*, with MIC values of 22.5, 1.7 and 1.1 µM, respectively. Compound **85** also showed weak cytotoxicity against A549, K562 and L-02 cell lines, with IC_50_ values of 8.1, 2.0 and 4.2 µM, respectively. The results showed that hydration and hydrogenation of the ∆^8^-double bond significantly reduce the cytotoxicity of aflatoxins, while the esterification at C-8 increases the cytotoxicity [76].

### 2.4. The Mechanism of Action of STC and AF

**Figure 12:**
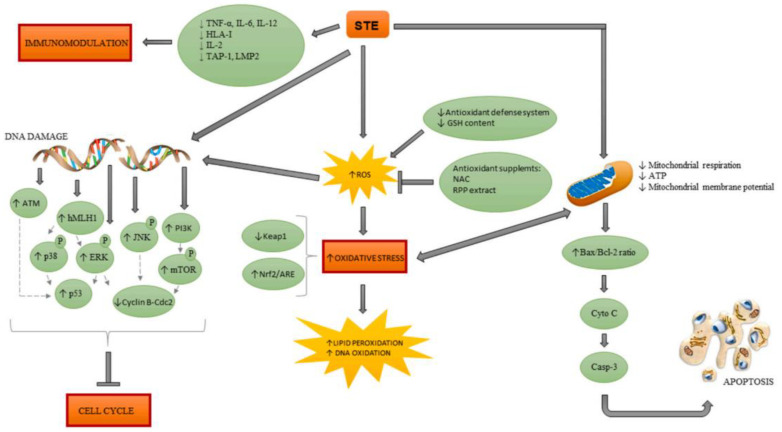
STC-induced molecular mechanisms of action [24].

Based on studies of in vitro models and in vivo studies, the reported metabolic transformations of STC and its ability to form DNA adducts and cause DNA damage appear to play a key role in STC-induced carcinogenesis. In addition, STC has been associated with diminished immune responses and dysregulation of adaptive immune system homeostasis, as well as with the induction of oxidative stress, apoptosis, mitochondrial dysfunction and the induction of oxidative stress, apoptosis, mitochondrial dysfunction and activation of specific pathways [24] (Figure 12).

This metabolite of AF is the major mutagen and carcinogen among the derived metabolites of AFs. Metabolic activation of AFB1, which occurs mainly in the liver. This compound forms covalent bonds with guanine residues in DNA, especially at N-7 guanine, leading to depurination and, hence, cancer. This active form binds to DNA and albumin in the serum, forming adducts that ultimately cause DNA damage [2,21,24].

## 3. Synthesis

### 3.1. Chemical Synthesis

Due to the unique skeletons of AFs and their risk to human health, numerous teams have been working on their total synthesis over the last 60 years. However, up to now, no reports have been found on the total synthesis of STCs.

#### 3.1.1. Total Synthesis of (±)-Aflatoxin B1

**Figure 13:**
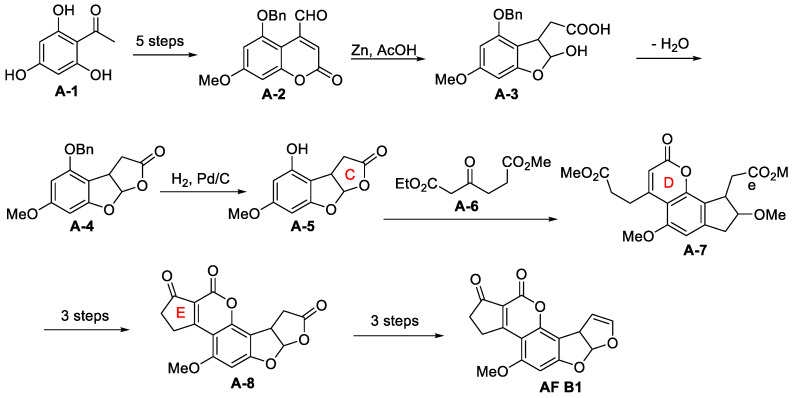
The total synthesis of (±)-Aflatoxin B1 [88].

The first total synthesis of AFB1 was completed in 1966, as shown in Figure 13 [87,88]. Compound **A-2** was obtained from **A-1** by a 5-step reaction including acylation, methylation and selective benzylation. In the presence of Zn/AcOH, the tricyclic skeleton **A-4** was efficiently constructed. The construction of the tricyclic framework was then completed by esterification, followed by the removal of the benzyl-protecting group, resulting in the tricyclic intermediate **A-5**.

Next, the D-ring was constructed by Peckman condensation reaction with *β*-keto ester **A-6**. Subsequently, in the presence of hydrochloric and acetic acids, the two ester groups underwent acetylmethyl hydrolysis, leading to the recycling of the C-ring. After activation of the carboxyl group, the E-ring was constructed by the Friedel-crafts reaction catalysed by AlCl_3_. AF B1 was synthesised by selective reduction of the C-ring, acylation of the hemiacetyl hydroxyl group and pyrolysis at 240 °C. The first total synthesis of AF B1 was accomplished in 13 steps with an overall yield of 0.9%.

#### 3.1.2. Total Synthesis of (±)-Aflatoxin B2

**Figure 14:**
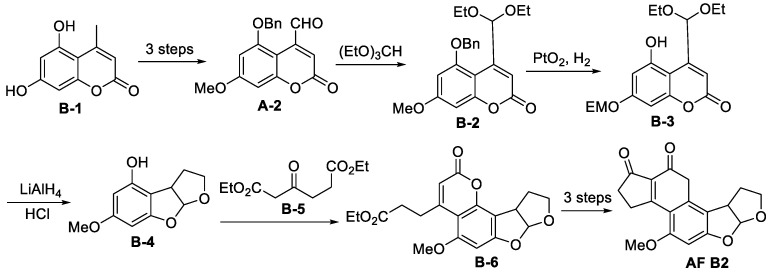
The total synthesis of (±)-Aflatoxin B2 [88].

In 1967, the first 10-step total synthesis of AFB2 was reported, as shown in Figure 14 [89,90]. Starting from **B-1**, coumarin intermediates **A-2** containing aldehyde groups were obtained by selective methylation, benzylation and allyl oxidation in the presence of SeO_2_. After the protection of the aldehyde group, the benzyl-protecting group was removed, and the double bond was hydrogenated in the presence of Adam’s catalyst. Subsequently, the ester group was reduced to alcohol in the presence of LiAlH_4_, and the aldehyde group was released in the presence of hydrochloric acid. Intramolecular acetals **B-4** were generated spontaneously and synthesised. The first synthesis of AFB2 was achieved by Peckman condensation and Friedel-process acylation.

#### 3.1.3. Total Synthesis of (±)-Aflatoxin M1

**Figure 15:**
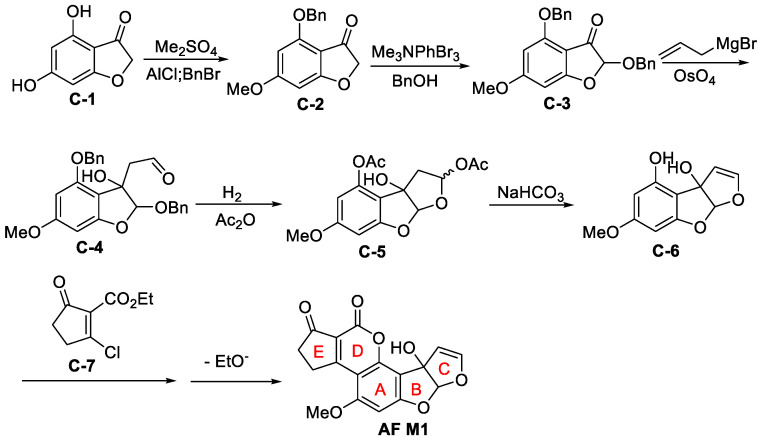
The total synthesis of (±)-Aflatoxin M1 [88].

In 1969, the first chemical total synthesis of AFM1 was reported, as shown in Figure 15 [88,90]. Based on the structural features of AFM1, dihydroxybenzofuranone **C-1** with a B-ring structure was used as a starting material to create conditions for the introduction of hydroxyl groups and to avoid the problem of constructing a B-ring. After dimethylation, selective demethylation and benzylation, the hydroxyl-protected benzofuranone **C-2** was obtained. The aldehyde **C-4** was then obtained by bromination (*α*-carbonyl), benzyl alcohol substitution, the addition of allyl magnesium bromide to the keto carbonyl group, and oxidative breakage of the double bond using osmium tetroxide. Removal of the two phenyl-protecting groups by hydrogenation, followed by acylation of the phenol and hemiacetyl hydroxyl groups and high-temperature pyrolysis (450 °C) to form a C-ring double bond.

The free phenolic hydroxyl group in the A-ring was released by hydrolysis in the presence of a weak base. In the presence of ZnCO_3_, the free phenolic compound **C-6** was reacted with chlorinated unsaturated cyclopentanone **C-7** to effectively construct the D, E-bicyclic compound. The specific reaction process was as follows. First, a 1, 4- addition reaction between the neighbouring phenolic hydroxyl group and the unsaturated carbon-based substrate **C-7** occurred smoothly under the catalysis of Zn ions. Next, the chloride anion was left as the leaving group to form the unsaturated carbonyl intermediate. Finally, in the presence of ZnCO_3_, the phenolic hydroxyl groups were esterified to form ethyl esters, thus completing the first total synthesis of AFM1. This method is a novel approach to the synthesis of AF.

#### 3.1.4. Total Synthesis of (±)-Aflatoxin G1

**Figure 16:**
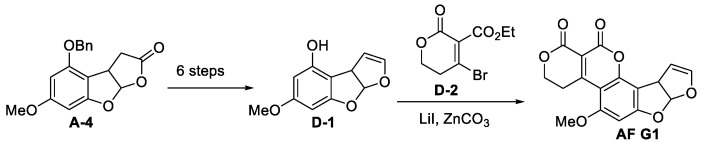
The total synthesis of (±)-Aflatoxin G1 [88].

The first total synthesis of AFG1 was accomplished in 1971 by a series of 1,4 addition, elimination and ester exchange reactions, as shown in Figure 16 [88,91]. Intermediate **D-1** was synthesised from intermediate **A-4** in six steps: DIBAL-H reduction, acylation, benzyl hydrogenation, phenolic hydroxyl acylation, hyperthermic decomposition, acylation of phenolic hydroxyl groups, high temperature (400 °C) pyrolysis and deacetylation. Finally, the D, E-bicyclic compound was formed in one step under reflux of ZnCO_3_ using the more active bromo-unsaturated caprolactone **D-2**.

## 4. Comprehensive Overview and Conclusions

STCs and AFs are the most important mycotoxins. Biologically, AFs are of concern to human health as they can be present as contaminants in food products [2]. AFs and STCs have attracted much attention in the last 70 years due to their unique skeletons and their risk to human health. This paper reviews the structures, biological activities, biosynthetic pathways, and chemical total synthesis of AFs and STCs.

Aflatoxins (AFs) are a specific group of mycotoxins primarily produced by toxigenic *Aspergillus* species, particularly *A. flavus* and *A. parasiticus*. They are highly toxic, mutagenic, carcinogenic, immunosuppressive compounds with severe detrimental effects on the human liver [2]. A better understanding of the mechanisms of gene regulation on aflatoxin biosynthesis will help us to identify natural inhibitors of fungal growth and aflatoxin formation.

A sample of STCs and their biological activities, sources are summarized in Table 1. Available data in the literature suggest that the cellular mechanisms of STC-induced toxicity include the induction of oxidative stress, mitochondrial dysfunction, apoptosis, and cell cycle arrest, as well as alteration of immune system function and activation of different signalling pathways. In addition, STC is genotoxic, forming DNA adducts and inducing DNA damage. Despite its high cytotoxicity, STC has not been subjected to risk assessment due to a lack of toxicity data, and studies on the toxicological effects of STC are continuing.

## Figures and Tables

**Table 1:** The compounds of Sterigmatocystins.

Compounds	Producing Strains	Habitats	Bioactivities	Refs.
1,2,3a,12c-tetrabydro-2-(3-chlorobenzoyloxy)-1,8-dihydroxy-6,11-dimethoxy-7*H*-furo[3′,2′:4,5]furo[2,3-c]-xanthen-7-one (**1**)	NR ^a^	Synthesis	NA ^b^	[25]
1,2,3a,12c-tetrabydro-2,8-dihydroxy-6,11-dimethoxy-7*H*-furo[3′,2′:4,5]furo[2,3-c]-xanthen-7-one (**2**)	NR	Synthesis	NA	[25]
1,2,3a,12c-tetrabydro-2,8-diacetoxy-6,11-dimethoxy-7*H*-furo[3′,2′:4,5]furo[2,3-c]-xanthen-7-one (**3**)	NR	Synthesis	NA	[25]
1,2,3a,12c-tetrabydro-5-bromo-2,8-diacetoxy-6,11-dimethoxy-7*H*-furo[3′,2′:4,5]furo[2,3-c]-xanthen-7-one (**4**)	NR	Synthesis	NA	[25]
sterigmatocystin-hemiacetal (**5**)	*Aspergillus* sp.	NR	NA	[26]
sterigmatocystin-ethoxyacetal (**6**)	*Aspergillus* sp.	NR	NA	[26]
*O*-methylsterigmatocystin-hemiacetal (**7**)	*Aspergillus* sp.	NR	NA	[26]
acyl-hemiacetal sterigmatocystin A (**8**)	*Aspergillus oryzae* M395	Sponge	NA	[27]
oxisterigmatocystin D (**9**)	*Aspergillus versicolor* D5	Alga-derived	NA	[28]
sterigmatocystin-1,2 oxide (**10**)	NR	NR	NA	[29]
sterigmatocystin-1,2 dihydrodiol (**11**)	NR	NR	NA	[29]
methylsterigmatocystin-1,2-oxide (**12**)	NR	NR	NA	[29]
methylsterigmatocystin-1,2-dihydrodiol (**13**)	NR	NR	NA	[29]
Aspertenol A (**14**)	*Aspergillus tennesseensis*	NR	NA	[30]
Aspertenol B (**15**)	*Aspergillus tennesseensis*	NR	Cytotoxicity Antiplasmodial activity	[30]
oxisterigmatocystins E (**16**)	*Botryotrichum piluliferum*	NR	Cytotoxicity Antiplasmodial activity	[31]
oxisterigmatocystins F (**17**)	*Botryotrichum piluliferum*	NR	Cytotoxicity Antiplasmodial activity	[31]
oxisterigmatocystins G (**18**)	*Botryotrichum piluliferum*	NR	Cytotoxicity Antiplasmodial activity	[31]
oxisterigmatocystins H (**19**)	*Botryotrichum piluliferum*	NR	Cytotoxicity Antiplasmodial activity	[31]
oxisterigmatocystin I (**20**)	*spergillus versicolor* A-21-2-7	South China Sea sediments	Antibacterial activity	[32,56]
terigmatocystin B (**21**)	*Aspergillus versicolor* 15XS43ZD-1	Sponge	NA	[33]
terigmatocystin C (**22**)	*Aspergillus versicolor* 15XS43ZD-1	Sponge	NA	[33]
oxisterigmatocystin A (**23**)	*Aspergillus versicolor* 15XS43ZD-1	Sponge	NA	[33]
oxisterigmatocystin B (**24**)	*Aspergillus versicolor* 15XS43ZD-1	Sponge	NA	[33]
oxisterigmatocystin C (**25**)	*Aspergillus versicolor* 15XS43ZD-1	Sponge	NA	[33]
oxisterigmatocystin J (**26**)	*Aspergillus nomius* NC06	sponge *Neopetrosia chaliniformis*	NA	[7]
oxisterigmatocystin K (**27**)	*Aspergillus nomius* NC06	Sponge *Neopetrosia chaliniformis*	NA	[7]
oxisterigmatocystin L (**28**)	*Aspergillus nomius* NC06	Sponge *Neopetrosia chaliniformis*	NA	[7]
sterigmatocystin B (**29**)	*Aspergillus nidulans*	Nyctanthes arbor-tristis	NA	[35]
*O*-methylsterigmatocystin (**30**)	*Aspergillus flavus*	NR	NA	[36]
sterigmatocystin (**31**)	*Aspergillus versicolor*	Irrigation of wild-strain spores	Cytotoxicity Antibacterial, Anti-inflammatory activity	[27,37,58,60]
5-methoxysterigmatocystin (**32**)	*Aspergillus versicolor*	Irrigation of wild-strain spores	Cytotoxicity Antibacterial, Anti-inflammatory activity	[27,37,58,59,60]
*O*-acetates (**33**)	*Aspergillus versicolor*	Irrigation of wild-strain spores	NA	[37]
aspertoxin acetate (**34**)	*Aspergillus flavus*	NR	NA	[38]
*O*-methyl-5-methoxysterigmatocystin (**35**)	*Aspergillus* sp.	NR	NA	[39]
*O*-acetyl-5-methoxysterigmatocystin (**36**)	*Aspergillus* sp.	NR	Cytotoxicity	[39]
5-methoxy-*O*-hydroxysterigmatocystin (**37**)	NR	Synthesis	NA	[25]
3a,12c-dihydro-9-allyl-8-hydroxy-6,11-dimethoxy-7*H*-furo[3′,2′:4,5]furo[2,3-c]xanthen-7-one (**38**)	NR	Synthesis	NA	[25]
3a,12c-dihydro-9-(or10)-nitro-8-hydroxy-6,11-dimethoxy-7*H*-furo[3′,2′:4,5]furo[2,3-c]xanth-en-7-one (**39**)	NR	Synthesis	NA	[25]
3a,12c-dihydro-9-amino-8-hydroxy-6,11-dimethoxy-7*H*-furo[3′,2′:4,5]furo[2,3-c] xanthen-7-one (**40**)	NR	Synthesis	NA	[25]
5,6-dimethoxysterigmatocystin (**41**)	*Aspergillus versicolor*	NR	NA	[40]
*O*-ethylsterigmatocystin (**42**)	*Aspergillus parasiticus*	NR	NA	[41]
*O*-propylsterigmatocystin (**43**)	*Aspergillus parasiticus*	NR	NA	[41]
*O*-butylsterigmatocystin (**44**)	*Aspergillus parasiticus*	NR	NA	[41]
*O*-penlylsterigmatocystin (**45**)	*Aspergillus parasiticus*	NR	NA	[41]
*O*-propenylsterigmatocystin (**46**)	*Aspergillus parasiticus*	NR	NA	[41]
*O*-acethlsterigmatocystin (**47**)	*Aspergillus parasiticus*	NR	NA	[41]
*O*-benzoylsterigmatocystin (**48**)	*Aspergillus parasiticus*	NR	NA	[41]
8-deoxysterigamoatocystin (**49**)	brown-black rotting mould fungus NRRL.22980	NR	NA	[42]
5-hydroxysterigmatocystin (**50**)	*Aschersonia coffeae* Henn.BCC 28712	Winged insect	Cytotoxicity	[43]
8-*O*-methoxysterigmatocystin (**51**)	*Aschersonia coffeae* Henn.BCC 28712	Winged insect	Cytotoxicity	[43]
demethylsterigmatocystin-1-(4-*O*-methyl)-*β*-L-arabinopyranose (**52**)	*Aschersonia coffeae* Henn.BCC 28712	Winged insect	Cytotoxicity Antiplasmodial activity	[43]
11-hydroxy-*O*-methylsterigmatocystin (**53**)	*Aspergillus parasiticus*	NR	NA	[44]
9-hydroxy-sterigmatocystin (**54**)	NR	NR	NA	[29]
12c-hydroxy-sterigmatocystin (**55**)	NR	NR	NA	[29]
11-hydroxy-sterigmatocystin (**56**)	NR	NR	NA	[29]
9,11-dihydroxy-sterigmatocystin (**57**)	NR	NR	NA	[29]
9,12c-dihydroxy-sterigmatocystin (**58**)	NR	NR	NA	[29]
11,12c-dihydroxy-sterigmatocystin (**59**)	NR	NR	NA	[29]
9-hydroxy-methylsterigmatocystin (**60**)	NR	NR	NA	[29]
9,12c-hydroxy-methylsterigmatocystin (**61**)	NR	NR	NA	[29]
N-0532B (**62**)	*Botryotrichum piluliferum*	Soil	NA	[31]
N-0532A (**63**)	*Botryotrichum piluliferum*	Soil	NA	[31]
*O*-demethyld-terigmatocystin (**64**)	*Aspergillus carneus*	Sponge	Antibacterial activity	[45,55]
*O*-acetyl-sterigmatocystin (**65**)	*Aspergillus nidulans*	Nyctanthes arbortristis	NA	[35]
dihydrosterigmatocystin (**66**)	*Aspergillus versicolor*	NR	Antibacterial activity	[37,55,56]
5-*O*-methyoxydihydrosterigmatocystin (**67**)	NA	Synthesis	NA	[37]
5-hydroxydihydrosterigmatocystin (**68**)	NA	Synthesis	NA	[37]
dihydrodemethylsterigmatocystin (**69**)	*Aspergillus versicolor*	NR	NA	[46]
5,6-dimethoxydihydrosterigmatocystin (**70**)	*Aspergillus versicolor*	NR	NA	[40]
11-hydroxy-*O*-methyldihydrosterigmatocystin (**71**)	*Aspergillus versicolor*	NR	NA	[44]
8-*O*-methyldihydrosterigmatocystin (**72**)	*Aschersonia coffeae* Henn. BCC 28712	Winged insect	NA	[43]

^a^ Not reported. ^b^ No activity reported in the reference.

## Data Availability

Not applicable.
